# Age‐related differences in mRNA vaccine immunogenicity and adjuvancy

**DOI:** 10.1111/imcb.70102

**Published:** 2026-03-12

**Authors:** Shivali Savita Chinni, Gemma S Trollope, Patrick Leung, Jonathan L McQualter, Chee Wah Tan, Linfa Wang, Leonard Lim, Adam K Wheatley, Jennifer A Juno, Stephen J Kent, Hareth A Al‐Wassiti, Colin W Pouton, Kylie M Quinn

**Affiliations:** ^1^ School of Health and Biomedical Sciences Royal Melbourne Institute of Technology (RMIT) University Bundoora VIC Australia; ^2^ Infectious Diseases Translational Research Programme, Yong Loo Lin School of Medicine National University of Singapore Singapore; ^3^ Department of Microbiology and Immunology, Yong Loo Lin School of Medicine National University of Singapore Singapore; ^4^ Duke‐NUS Medical School Singapore Singapore; ^5^ Monash Institute of Pharmaceutical Sciences (MIPS) Monash University Parkville VIC Australia; ^6^ Department of Microbiology and Immunology, The Peter Doherty Institute for Infection and Immunity University of Melbourne Melbourne VIC Australia; ^7^ Melbourne Sexual Health Centre and Department of Infectious Diseases, Alfred Health, Central Clinical School Monash University Melbourne VIC Australia; ^8^ Department of Biochemistry, Biomedicine Discovery Institute Monash University Clayton VIC Australia

**Keywords:** adjuvancy, aging, immunogenicity, mouse, mRNA vaccine

## Abstract

Older people mount poorer adaptive immune responses to mRNA vaccines, leaving them more vulnerable to infection with SARS‐CoV‐2. To design better mRNA vaccines for older people, we need to understand how aging alters mechanisms of adjuvancy that shape immunogenicity. To first define age‐related changes in immunogenicity, we vaccinated young (< 5 months old) and aged (> 18 months old) C57BL/6 mice with an mRNA vaccine encoding the SARS‐CoV‐2 spike protein. T cell responses were markedly reduced in aged mice at peak and memory timepoints, using intracellular cytokine staining or activation‐induced marker assays. Spike and receptor‐binding domain binding and neutralizing antibody titers were also markedly reduced in aged mice, consistent with deficits seen in older humans. To define age‐related changes in adjuvancy mechanisms, we vaccinated young and aged mice with mRNA vaccines loaded with DiD lipid dye or mScarlet mRNA, then tracked dendritic cell (DC) numbers, phenotype, vaccine uptake, antigen expression, and activation, as well as local and systemic cytokine production. DC numbers in the draining lymph nodes (dLN) were dramatically reduced before and early after vaccination in aged compared to young mice, with delayed recruitment of DCs to the dLN. Vaccine uptake was not impacted by age, but the frequency of DCs expressing antigen increased with age and DC activation decreased with age. Aging accelerated the expression of some cytokines (IL‐1α, IL‐6), while delaying others (IFNγ, MCP‐1) in dLNs and sera. This illustrates that aging impairs multiple adjuvancy mechanisms but mRNA vaccine strategies that address these age‐related deficits could improve responses in older people.

## Graphical abstract

Older people often respond poorly to mRNA vaccines, with reduced T and B cell responses, but they also bear more morbidity and mortality for infectious diseases. This paper maps age‐related changes in mechanisms of adjuvancy that can shape the immunogenicity of mRNA vaccine in a mouse model. Created in BioRender.

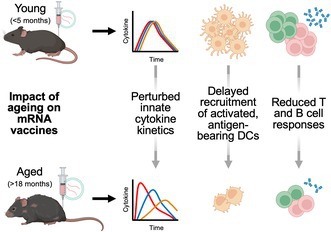

## INTRODUCTION

mRNA vaccines have had a remarkable trajectory in recent years. Clinical SARS‐CoV‐2 vaccines are now well‐established,^1^, ^2^, ^3^ with new vaccines for other infectious diseases at advanced stages of testing or in development^4^, ^5^ and even cancer vaccines are in the clinic.^6^, ^7^ However, like many other vaccine modalities, mRNA vaccines often generate more modest responses in older individuals.

The impact of aging on mRNA vaccine immunogenicity and/or efficacy has been examined in multiple studies of SARS‐CoV‐2 vaccines. Phase 1–3 clinical trials^1^, ^2^, ^3^ and post‐approval studies^8^, ^9^, ^10^, ^11^, ^12^ have reported reduced B and/or T cell responses in older vaccinees, particularly after the first dose. Some studies reported that booster doses can increase or even equalize adaptive responses at peak in younger and older vaccinees.^11^, ^13^ However, responses still wane,^14^, ^15^ and vaccine advisory bodies now generally recommend that older people receive booster doses every 6–12 months. Older people clearly have reduced T and B cell responses to mRNA vaccines, but this demographic also has the highest rates of morbidity or mortality with infectious diseases^1^, ^2^, ^3^, ^4^, ^5^ or cancers^6^, ^7^ that are being targeted by new or emerging mRNA vaccines. If mRNA vaccines could be tailored for the specific needs of an older immune system, vaccine immunogenicity and efficacy could be improved for older patients. To identify strategies to improve vaccine design, we first need to precisely define how aging alters mechanisms of mRNA vaccine adjuvancy.

Adjuvancy is defined as any mechanism that promotes vaccine‐induced adaptive immunity, in particular, a vaccine's capacity to (i) support robust antigen signaling and/or (ii) trigger specific innate immune signals.

Antigen signals are controlled principally by vaccine uptake and antigen processing and presentation by relevant antigen presenting cells (APCs). For mRNA vaccines, the mRNA/lipid nanoparticle (LNP) is endocytosed, ionizable lipids in the LNP mediate escape of the mRNA cargo from the endosome into the cytosol, and the mRNA is used as a template for translation of the target antigen.^16^ Mouse and non‐human primate models have highlighted that both non‐immune and immune cells can take up mRNA/LNPs after vaccination, including APCs at the vaccine site, in dLNs and at more distal sites with lower efficiency.^17^, ^18^ Despite broad biodistribution, it is not clear if all APCs are equally capable of antigen presentation after mRNA vaccination. Two APC subsets, known as conventional DC1s (cDC1s) and Langerhans cells (LCs), were shown to be essential for optimal priming of T and B cell responses after vaccination with a proprietary mRNA/LNP developed by Acuitas,^19^ but any requirement for specific APCs with Comirnaty and Spikevax formulations is not published.

Innate immune signals are driven by both the mRNA and LNP. The mRNA or residual contaminants from mRNA production can trigger multiple pattern recognition receptors (PRRs), including TLR3, 7 and 8, and RIG‐I and MDA‐5, to drive type I interferon (IFN) production, which can potentially inhibit antigen expression. Type I IFN production can be attenuated and antigen expression can be maintained if certain steps are taken during mRNA production; using nucleoside analogs,^20^ ensuring appropriate 5′ capping, performing careful template design, and clean‐up to avoid contaminants to limit triggering of PRRs.^16^ However, some type I IFN signaling is still essential for CD8 T cell responses and optimal for B cell responses after mRNA vaccination.^21^, ^22^ The immunostimulatory capacity of LNPs can change substantially with the use of different ionizable lipids, but clinically relevant LNPs can trigger the NLRP3 inflammasome^23^ presumably by disrupting the cell membrane^24^ and can trigger other TLRs such as TLR4 in monocyte‐derived cell‐lines.^25^ Clinically relevant LNPs have been shown to stimulate production of IL‐1α and IL‐1β, which then drives IL‐6 production, which all correlate with more robust CD4 T follicular helper and B cell responses after mRNA vaccination.^19^, ^23^

Several *in vitro* and *in vivo* studies have explored the impact of aging on mRNA/LNP vaccines. Connors and colleagues exposed human DCs and monocyte‐derived DCs to empty LNPs (eLNPs) *in vitro* and observed age‐related reductions in activation markers (CD40, CD83, 4‐1BBL) or cytokines (IFNγ), with heightened production of TGF‐β2.^26^ Brook and colleagues co‐incubated mRNA/LNPs with peripheral blood from younger (18–50 yo) and older (> 65 yo) vaccinated donors and observed age‐related blunting of T_H_1‐related cytokine production (IFNγ, TNF, and CXCL10) with similar results seen in a mouse model.^27^ Finally, Nanishi and colleagues vaccinated young and aged Balb/c mice and observed clear age‐related deficits in adaptive immune responses and protection from SARS‐CoV‐2 infection.^28^ These studies have partially explored mechanisms of adjuvancy *in vitro* with aged human samples or immunogenicity *in vivo* with aged mouse models, but the field lacks a comprehensive *in vivo* assessment of age‐related impacts on both immunogenicity and adjuvancy of mRNA vaccines.

In this study, we validated the impact of aging on mRNA vaccine immunogenicity in a C57BL/6 mouse model and then used this model to define age‐related changes in key mechanisms of adjuvancy. We assessed the impact of aging on local and systemic inflammatory cytokine production and on DC number, phenotype and activation in the dLN and spleen, vaccine uptake and antigen expression in the dLN. We observed a dramatic deficit in DC numbers in the dLN but not the spleen, delayed DC recruitment, retention of antigen‐bearing DCs and a dysregulation of innate cytokines in the dLN. This data therefore provides clear targets to modulate, with the aim of improving T and B cell immune responses to mRNA vaccines in older people.

## RESULTS

### Aging impairs adaptive immunity in mice after mRNA vaccination

To understand how aging impacts mRNA vaccine immunogenicity in a mouse model, we first validated that mice exhibit similar age‐related deficits to humans.^8^, ^9^ We vaccinated young (< 5 months) and aged (> 18 months) C57BL/6 mice with an mRNA vaccine (Supplementary figure 2a). The vaccine encoded the SARS‐CoV‐2 Wuhan strain spike protein and used a similar LNP to the Comirnaty mRNA vaccine formulation. We used a similar primary dosing schedule to clinical mRNA vaccines, with two doses administered at day 0 and day 28. To assess antigen‐specific T cell responses, we took spleen samples at day 42 (14 days post‐boost) for peak responses and day 93 (65 days post‐boost) for memory responses and performed ICS and AIM assays. To assess antigen‐specific B cell responses, we took serum samples at days 21 (peak post‐prime), 28 (time of boost), 42 (peak post‐boost) and 93 (memory) and performed ELISA assays of total spike‐ or RBD‐specific IgG and a surrogate neutralization assay.

**Figure 1:**
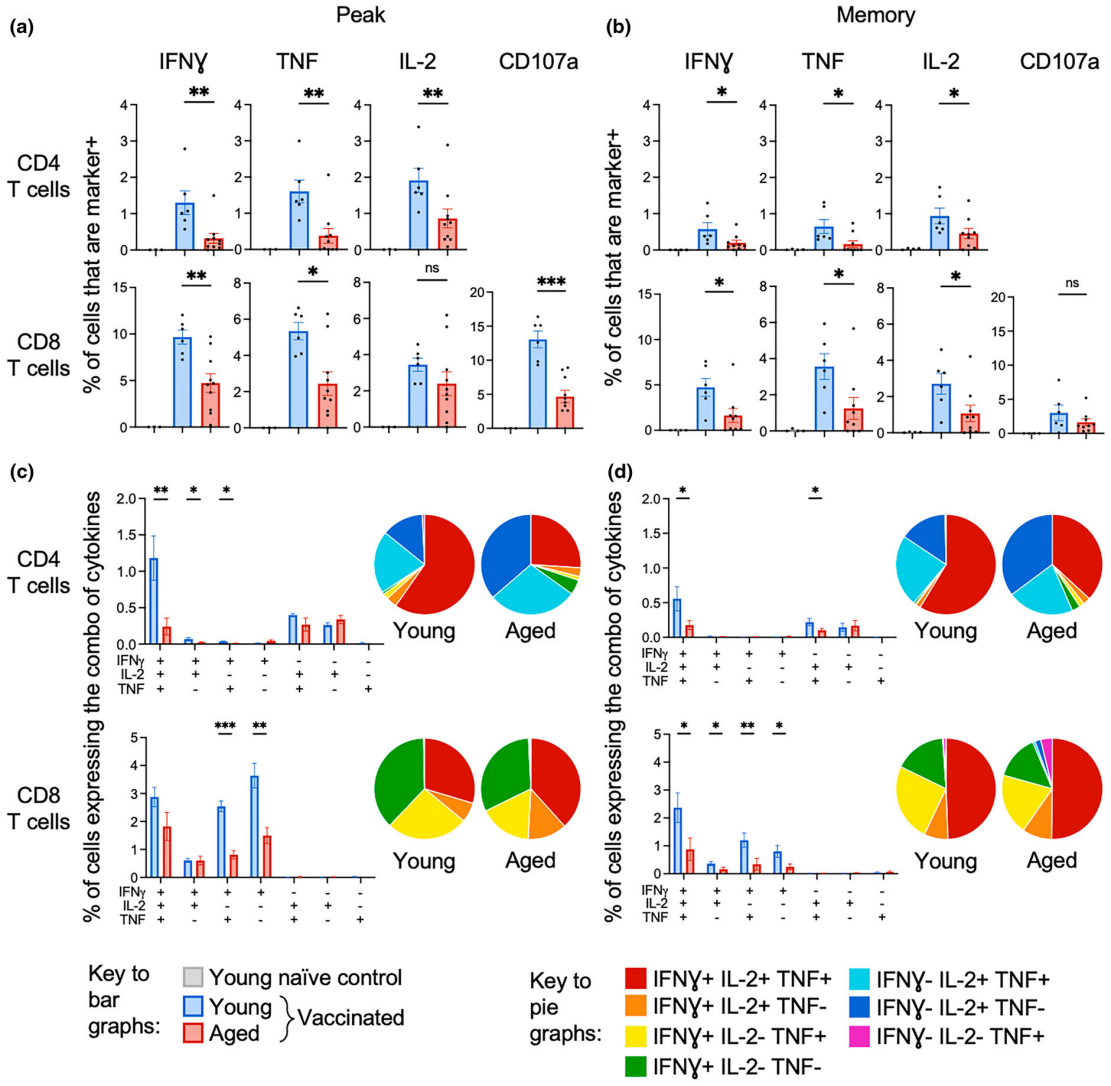
Aging leads to a decrease in antigen‐specific T cell cytokine responses after mRNA vaccination. **(a)** Frequency of CD4 (top) or CD8 (bottom) T cells that produce spike‐specific IFNγ, IL‐2, or TNF or CD8 T cells that are spike‐specific and CD107a^+^ at peak (day 42 after vaccination). **(b)** Frequency of CD4 (top) or CD8 (bottom) T cells that produce spike‐specific IFNγ, IL‐2, or TNF or CD8 T cells that are spike‐specific and CD107a^+^ at memory (day 93 after vaccination). **(c)** Frequency and proportion of CD4 (top) or CD8 (bottom) T cells that produce any combination of spike‐specific IFNγ, IL‐2, and/or TNF at peak. **(d)** Frequency and proportion of CD4 (top) or CD8 (bottom) T cells that produce any combination of spike‐specific IFNγ, IL‐2, and/or TNF at memory. Reported frequencies are background subtracted, with CD4 T cells stimulated with a peptide library and CD8 T cells stimulated with an immunodominant peptide for spike. Bars indicate mean, error bars indicate SEM, and symbols indicate individual mice (*n* = 4 (young naïve), 6 (young vaccinated) or 10 (aged vaccinated)). * indicates *P* ≤ 0.05, ** indicates *P* ≤ 0.01, *** indicates *P* ≤ 0.001, using a Mann–Whitney *U* test. Data are representative of 2 independent experiments.

The frequency of CD4 or CD8 T cells producing IFNγ or TNF was significantly lower after vaccination for aged compared to young mice at both peak and memory timepoints (Figure 1a, b). IL‐2 production was also significantly reduced in aged mice at peak and/or memory and CD107a staining was significantly reduced in CD8 T cells in aged mice at peak (Figure 1a, b). These findings are consistent with human data, which showed age‐related reductions in spike‐specific IFNγ and IL‐2 responses in T cells after SARS‐CoV‐2 mRNA vaccination.^8^, ^10^, ^11^ The ability of antigen‐specific T cells to express multiple cytokines (particularly IFNγ, IL‐2, or TNF) is termed “multifunctionality” and these cells are regarded as higher quality, as they produce more of each cytokine on a per cell basis, have more functional plasticity, and higher memory potential.^29^ For CD4 T cells, the absolute frequency and proportion of cells that were multifunctional were significantly lower in aged compared to young mice at both peak and memory timepoints (Figure 1c, d). For CD8 T cells, the absolute frequency of cells that were multifunctional was significantly lower in aged mice at the memory timepoint, but proportions were well‐maintained at both peak and memory (Figure 1c, d). A reduction of multifunctional T cells with increased age has been observed in other murine studies^30^ and our data show that T cell responses after mRNA vaccination are both numerically and functionally impaired by increasing age.

**Figure 2:**
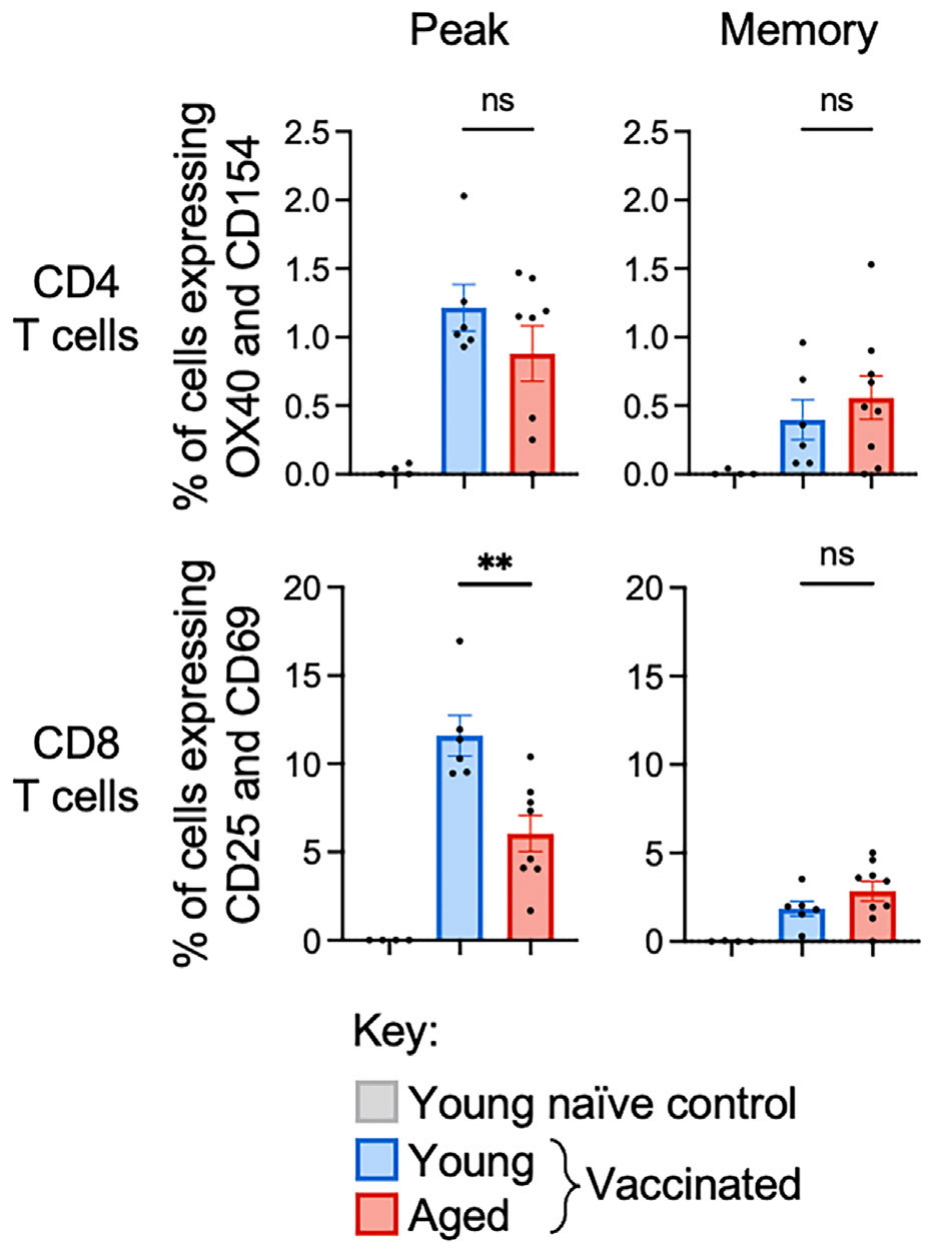
Aging leads to a decrease in antigen‐specific activation‐induced marker expression on T cells early after mRNA vaccination. The frequency of CD4 T cells co‐expressing CD154 and OX40 (top) or CD8 T cells co‐expressing CD25 and CD69 (bottom) at day 42 (peak; left) or day 93 (memory; right). Reported frequencies are background subtracted, with CD4 T cells stimulated with a peptide library and CD8 T cells stimulated with an immunodominant peptide for spike. Bars indicate mean, error bars indicate SEM, and symbols indicate individual mice (*n* = 4 (young naïve), 6 (young vaccinated) or 10 (aged vaccinated)). ns indicates not significant, * indicates *P* ≤ 0.05, ** indicates *P* ≤ 0.01, *** indicates *P* ≤ 0.001, using a Mann–Whitney *U* test. Data are representative of 2 independent experiments.

To complement the ICS assay‐based assessment of spike‐specific T cell responses, we also performed an AIM assay. We used CD154 and OX40 co‐expression to identify spike‐specific CD4 T cells and CD69 and CD25 co‐expression to identify spike‐specific CD8 T cells. The frequency of CD4 T cells that co‐expressed CD154 and OX40 trended lower, and the frequency of CD8 T cells that co‐expressed CD25 and CD69 was significantly lower in aged mice at peak (Figure 2). However, the frequency of spike‐specific CD4 or CD8 T cells co‐expressing activation markers did not differ across young and aged mice at memory (Figure 2). We noted that aged mice had markedly higher frequencies of unstimulated CD4 T cells expressing CD154 and/or OX40 and CD8 T cells expressing CD25 and/or CD69 (Supplementary figure 3). Given the elevated background expression of these activation markers, we caution that AIM assays may lack sensitivity for measurement of antigen‐specific T cell responses in aged mice.

**Figure 3:**
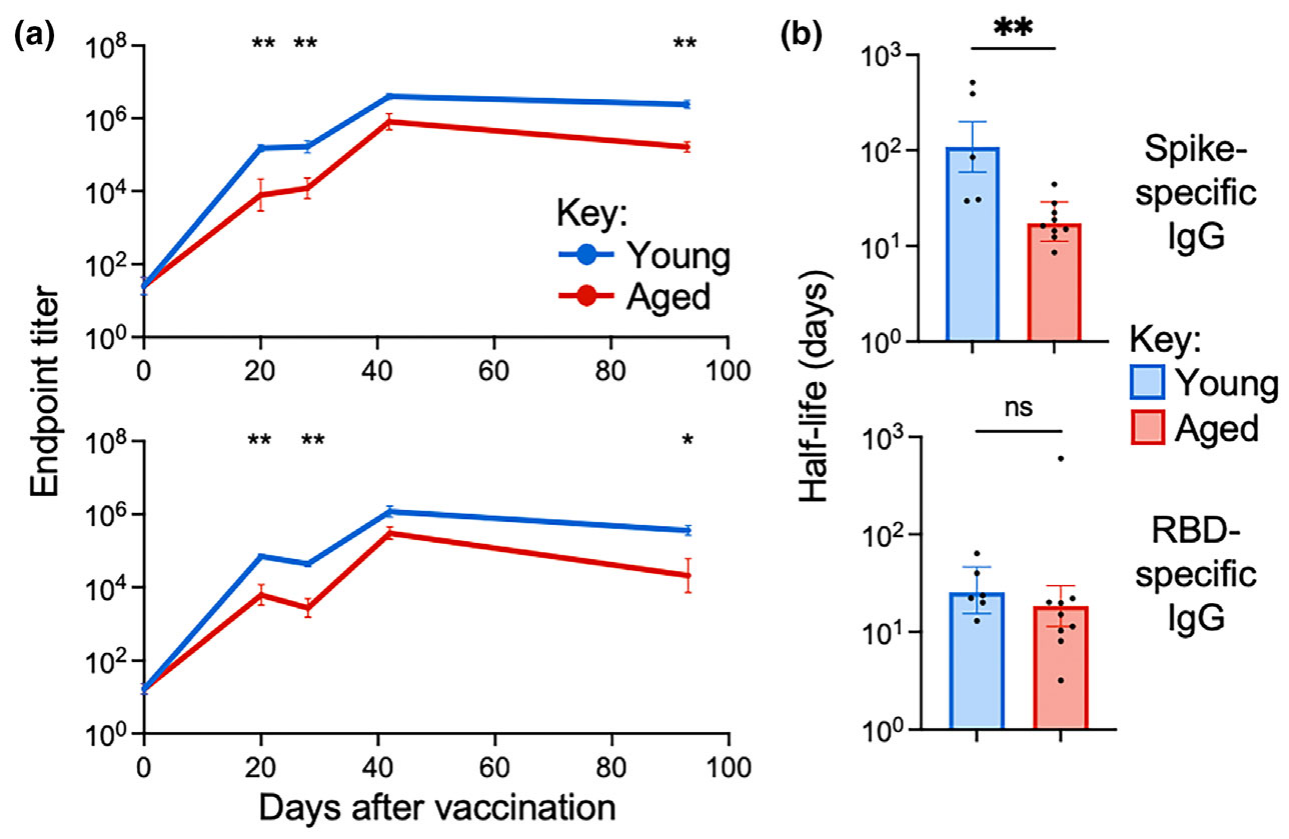
Aging leads to a decrease in B cell responses after mRNA vaccination in mice. **(a)** Endpoint titers of total IgG specific for either full‐length spike protein or the RBD of spike protein in sera from young or aged mice at days 0, 21, 28, 42 and 93 after mRNA vaccination. **(b)** Average half‐life in days for IgG directed against full‐length spike protein or the RBD of spike protein in sera from young or aged mice after mRNA vaccination. On line graphs, symbols indicate mean, error bars indicate SEM (*n* = 6 (young vaccinated) or 10 (aged vaccinated)). On bar graphs, bars indicate mean, error bars indicate SEM (*n* = 6 (young vaccinated) or 10 (aged vaccinated)). ns indicates not significant, * indicates *P* ≤ 0.05, ** indicates *P* ≤ 0.01, *** indicates *P* ≤ 0.001, using a Mann–Whitney *U* test with Bonferroni–Dunn correction for multiple comparisons, where appropriate. Data are representative of 2 independent experiments.

When antibody titers were assessed, aging led to significantly lower levels of total IgG specific for full‐length spike and RBD at days 21, 28 and 93 (Figure 3a, b). Titres waned from peak to memory timepoints in both young and aged mice (Figure 3a, b), consistent with human pre‐ and post‐approval studies.^1^, ^2^, ^3^, ^8^, ^9^ When the half‐life of antigen‐specific total IgG titers was calculated as a measure of waning, spike‐specific IgG was consistently significantly shorter for aged (16 days) compared to young (69.97 days) mice and consistently trended shorter for RBD‐specific IgG (15.11 days vs 22.96 days) (Figure 3c). This is consistent with some post‐approval vaccine studies in humans that have suggested that aging accelerates waning of antibody titers.^11^ Few studies have quantified SARS‐CoV‐2‐specific antibody half‐life in serum, and those that have do not see a significant difference,^13^ but our data suggests that the rate of decay for antibody titers should be monitored in older populations after vaccinations.

When surrogate virus neutralization titers were measured using a bead‐based assay, significantly lower titers were observed in sera harvested at day 93 from aged as compared to young mice for the original Wuhan variant and multiple additional variants (Supplementary figure 4). This suggests that the age‐related impairment of IgG titers causes a functional defect in B cell responses, reducing the capacity to neutralize both antigen‐matched viruses and variants.

Collectively, these results demonstrate that the mouse model recapitulates age‐related deficits in T and B cell responses seen in humans vaccinated with current clinical mRNA vaccines. We then used the aged mouse model to define how aging alters early events after mRNA vaccination – specifically innate cytokine signals and antigen signals – to potentially limit subsequent T and B cell responses.

### Aging perturbs local and systemic production of innate cytokines after mRNA vaccination

**Figure 4:**
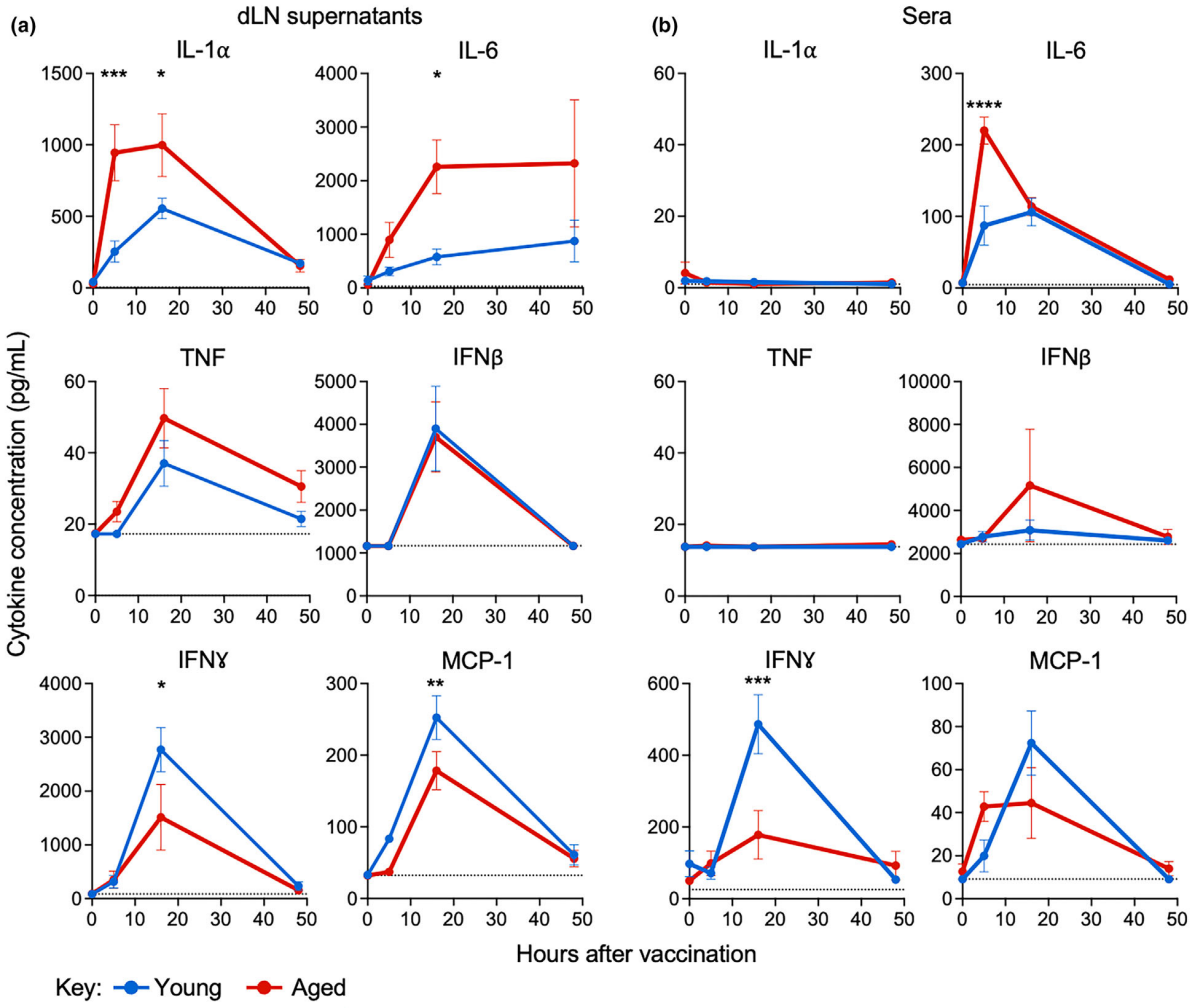
Aging alters local and systemic innate cytokine production after mRNA vaccination. Concentration of IL‐1α, IL‐6, TNF, IFNβ, IFNγ, and MCP‐1 in **(a)** supernatants from dLN or **(b)** sera harvested at 0, 5, 16, and 48 h after mRNA vaccination. Symbols indicate mean, error bars indicate SEM. (*n* = 4–6 young vaccinated or aged vaccinated). ns indicates not significant, * indicates *P* ≤ 0.05, ** indicates *P* ≤ 0.01, *** indicates *P* ≤ 0.001, using a two‐way ANOVA test with Sidak's correction for multiple comparisons. Data are representative of 2 independent experiments.

To assess how aging impacts inflammatory cytokine production early after mRNA vaccination, we vaccinated young and aged mice with one dose of the SARS‐CoV‐2 Spike‐encoding mRNA vaccine, then harvested dLNs and sera at 0, 5, 16, and 48 h after vaccination (Supplementary figure 2b). After harvest, dLNs were homogenized in cRPMI, cultured for a further 5 h, and supernatants were assayed for cytokines. The dLN supernatants and sera were assayed for 13 different cytokines using the Legendplex 13‐plex mouse inflammation panel. Multiple cytokines were predominantly below the limit of detection for the assay locally and systemically (IL‐1β, IL‐10, IL‐12p70, IL‐17, IL‐23, IL‐27, and GM‐CSF). However, substantial production of IL‐1α, IL‐6, TNF, IFNβ, IFNγ, and MCP‐1 was detected at one or more timepoints locally and/or systemically (Figure 4). The kinetics of these cytokines in young mice after mRNA vaccination were comparable to previously published data,^21^ but aging had mixed effects on these kinetics.

Production of IL‐1α and IL‐6 was significantly accelerated and increased locally (Figure 4a), and IL‐6 was also accelerated and increased systemically (Figure 4b) with increased age. Given that IL‐1α and IL‐6 tend to correlate with more robust B cell responses after mRNA vaccination,^23^ increased expression of these cytokines is not consistent with the decreased antibody titers seen in aging mice. Production of TNF and IFNβ was not impacted by age locally (Figure 4a) or systemically (Figure 4b). Given that type I IFN is essential for CD8 T cell responses and optimal for B cell responses after mRNA vaccination,^21^, ^22^ the age‐related reduction in CD8 T cell responses and antibody titers does not appear to be due to changes in IFNβ production. Finally, production of IFNγ and MCP‐1 was significantly decreased locally (Figure 4a) and/or systemically (Figure 4b). IFNγ production is driven by sensing of type I IFNs, IL‐12, and itself, and IFNγ early after mRNA vaccination is mainly generated by NK cells.^21^ MCP‐1 production is driven by sensing of IFNβ, IL‐6, and TNF, and it is typically generated by macrophages but also potentially other cells such as fibroblasts and endothelia. Age‐related reductions in IFNγ production by NK cells and MCP‐1 production by macrophages have been seen in humans or mouse models,^31^, ^32^ so our data are consistent with prior work, and this could contribute to reduced adaptive responses to mRNA vaccines. Given that we did not enumerate leukocyte subsets at each timepoint, it is not clear whether changes in cytokine production are due to numeric fluctuations in leukocytes that predominantly produce a given cytokine or their underlying function. Regardless of the source or the directionality of change in an individual cytokine, our dataset clearly shows that aging causes dramatic shifts in production of cytokines relative to one another both locally and systemically.

### Aging reduces DC numbers and delays DC recruitment after mRNA vaccination

To assess how aging alters DC numbers and subsets, migration to the dLN, vaccine uptake by DCs, antigen expression by DCs, and costimulatory molecule expression, we vaccinated young and aged mice with mRNA vaccines that were either loaded with a DiD lipid dye to track vaccine uptake or that contained an mRNA encoding the mScarlet fluorescent protein to track antigen expression (Supplementary figure 2c). We harvested dLNs at 0, 5, 16 and/or 48 h after vaccination, performed magnetic bead enrichment to isolate DCs, and then measured DC numbers, DiD staining, mScarlet staining, and expression of CD86 by flow cytometry. We tracked these parameters in total DCs and in three DC subsets: cDC1s, cDC2s, and plasmacytoid DCs (pDCs) (Supplementary figure 1c, d).

**Figure 5:**
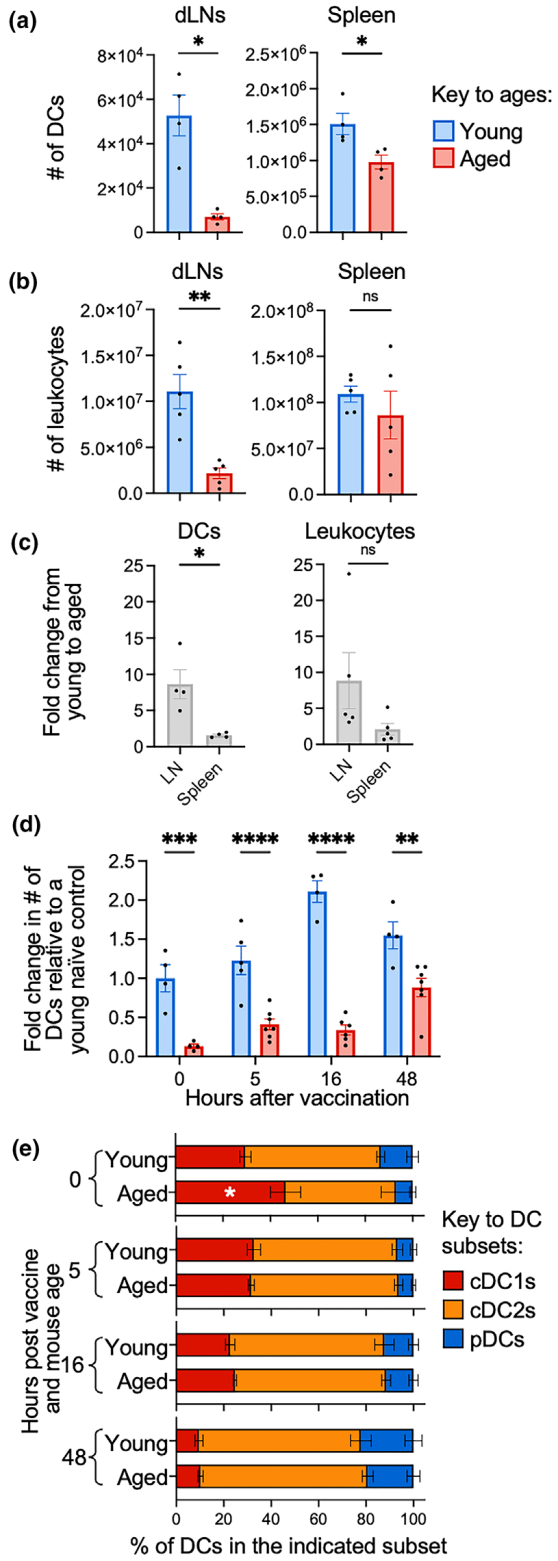
Aging reduces DC numbers and delays DC recruitment to the dLN after mRNA vaccination. **(a)** Total DC numbers in dLNs and spleen in young and aged naïve mice. **(b)** Total number of live leukocytes in dLNs and spleen in young and aged naïve mice. **(c)** Fold change from average young to aged mice in the dLN vs spleen for DC or leukocyte numbers. **(d)** Fold change in DC numbers (relative to an average young naïve control) in young and aged vaccinated mice and **(e)** the frequency of total DCs that are each subset at 0, 5, 16 and 48 h after mRNA vaccination. Symbols indicate mean, error bars indicate SEM. (*n* = 4 (young vaccinated) or 6 (aged vaccinated)). ns indicates not significant, * indicates *P* ≤ 0.05, ** indicates *P* ≤ 0.01, *** indicates *P* ≤ 0.001, using a Mann–Whitney *U* test (A/B) or two‐way ANOVA test with Sidak's correction for multiple comparisons (C/D). Data are representative of at least 2 independent experiments.

We first evaluated how aging impacted DC numbers prior to vaccination. We observed a dramatic age‐related reduction in DCs in the dLN of naïve mice but only a modest age‐related reduction in the spleen (Figure 5a). The overall cellularity of dLNs was also dramatically reduced, while the spleen only trended down (Figure 5b) and average age‐related fold reduction in DC numbers and leukocyte numbers was highly proportional across tissues (~8.6‐fold for DCs and ~8.8‐fold for leukocytes in the dLN; ~1.6‐fold for DCs and ~2.1‐fold for leukocytes in the spleen) (Figure 5c). We then tracked DC numbers after mRNA vaccination and observed that DC numbers started to increase from 5 h after vaccination and peaked between 16 and 48 h in young mice, while DC numbers remained low in aged mice until 48 h after vaccination (Figure 5d). This suggests that there is an age‐related lag in DC recruitment to dLNs, which is consistent with reports of reduced DC migration with increasing age.^33^, ^34^, ^35^ Of note, aging modestly increased the frequency of cDC1s in naïve mice and did not markedly alter the composition of DC subsets at any timepoint assessed after vaccination (Figure 5e), so the age‐related deficit in DC numbers impacted all major DC subsets analyzed.

### Aging does not impact mRNA vaccine uptake

**Figure 6:**
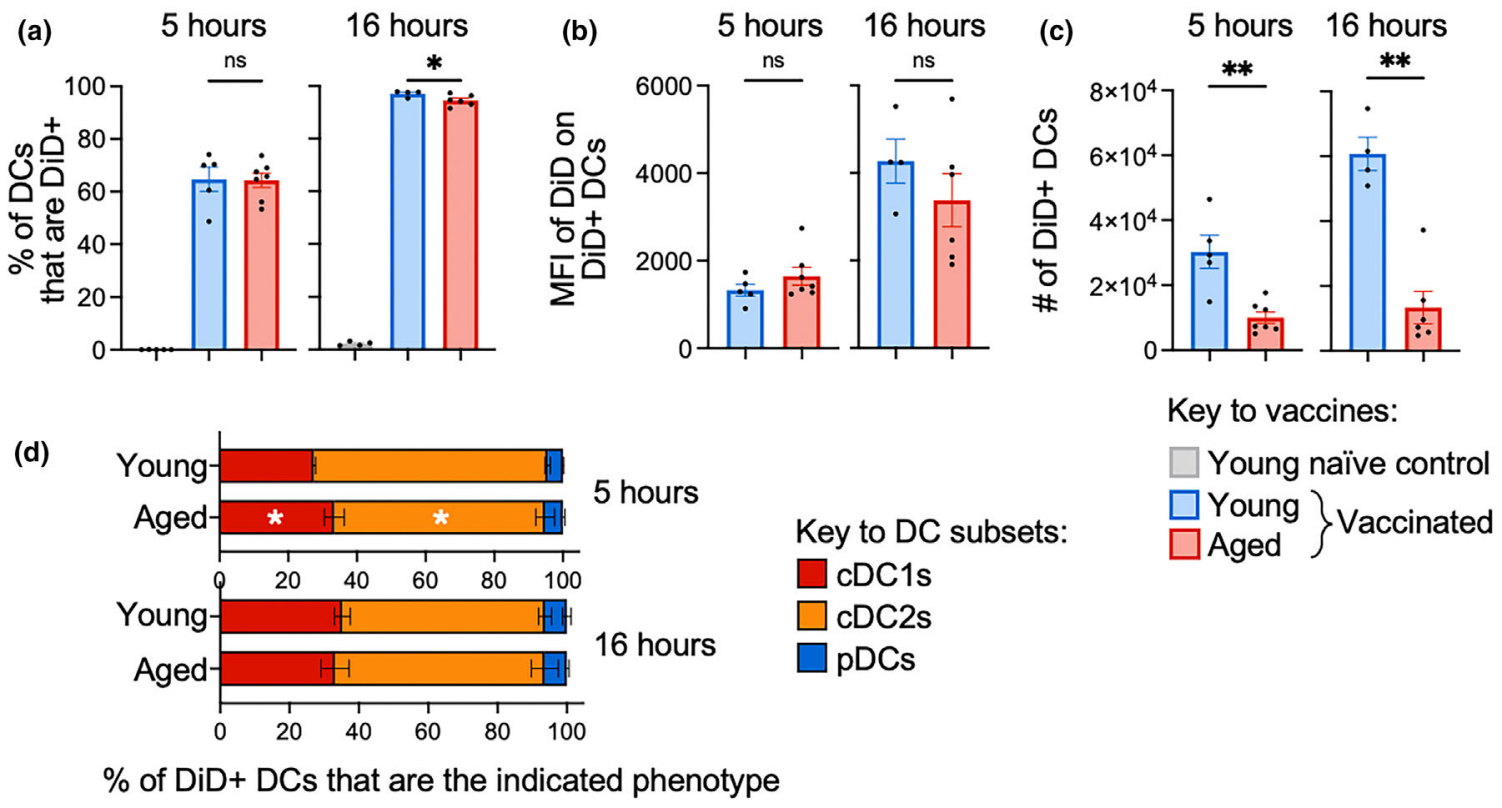
Aging does not impact uptake of mRNA vaccines. **(a)** The frequency of DCs that are DiD^+^, **(b)** the MFI of DiD on DiD^+^ DCs, **(c)** absolute numbers of DiD^+^ DCs and **(d)** the frequency of DiD^+^ DC in dLN that are each subset at 5 and 16 h after mRNA vaccination in young and aged vaccinated mice. Symbols indicate mean, error bars indicate SEM. (*n* = 4 (young vaccinated) or 6 (aged vaccinated). ns indicates not significant, * indicates *P* ≤ 0.05, ** indicates *P* ≤ 0.01, *** indicates *P* ≤ 0.001, using a Mann–Whitney *U* test. Data are representative of at least 2 independent experiments.

We then evaluated how aging impacted vaccine uptake by DCs in the dLN. The frequency of DCs that had taken up the vaccine was unaffected by age at 5 h after vaccination and modestly reduced in aged mice at 16 h after vaccination (Figure 6a). The median fluorescence intensity (MFI) of DiD staining in DCs that had taken up the vaccine was unaffected (Figure 6b), suggesting the amount of vaccine taken up per cell is not impacted by age. However, given the overall deficit in DC numbers noted previously (Figure 5a), the absolute number of DiD^+^ DCs recovered from the dLNs was substantially lower in aged as compared to young mice (Figure 6c). When DC subset composition was assessed, the frequencies of subsets within DiD^+^ DCs (Figure 6d) did not differ from total DCs at a matched timepoint (Figure 5d), which suggests that the mRNA vaccine is not preferentially taken up by specific DC subsets. The frequencies of subsets within DiD^+^ DCs also did not differ across young and aged mice (Figure 6d), which demonstrates aging does not alter uptake of mRNA vaccine by distinct DC subsets.

### Aging increases the frequency of antigen expressing DCs after mRNA vaccination

**Figure 7:**
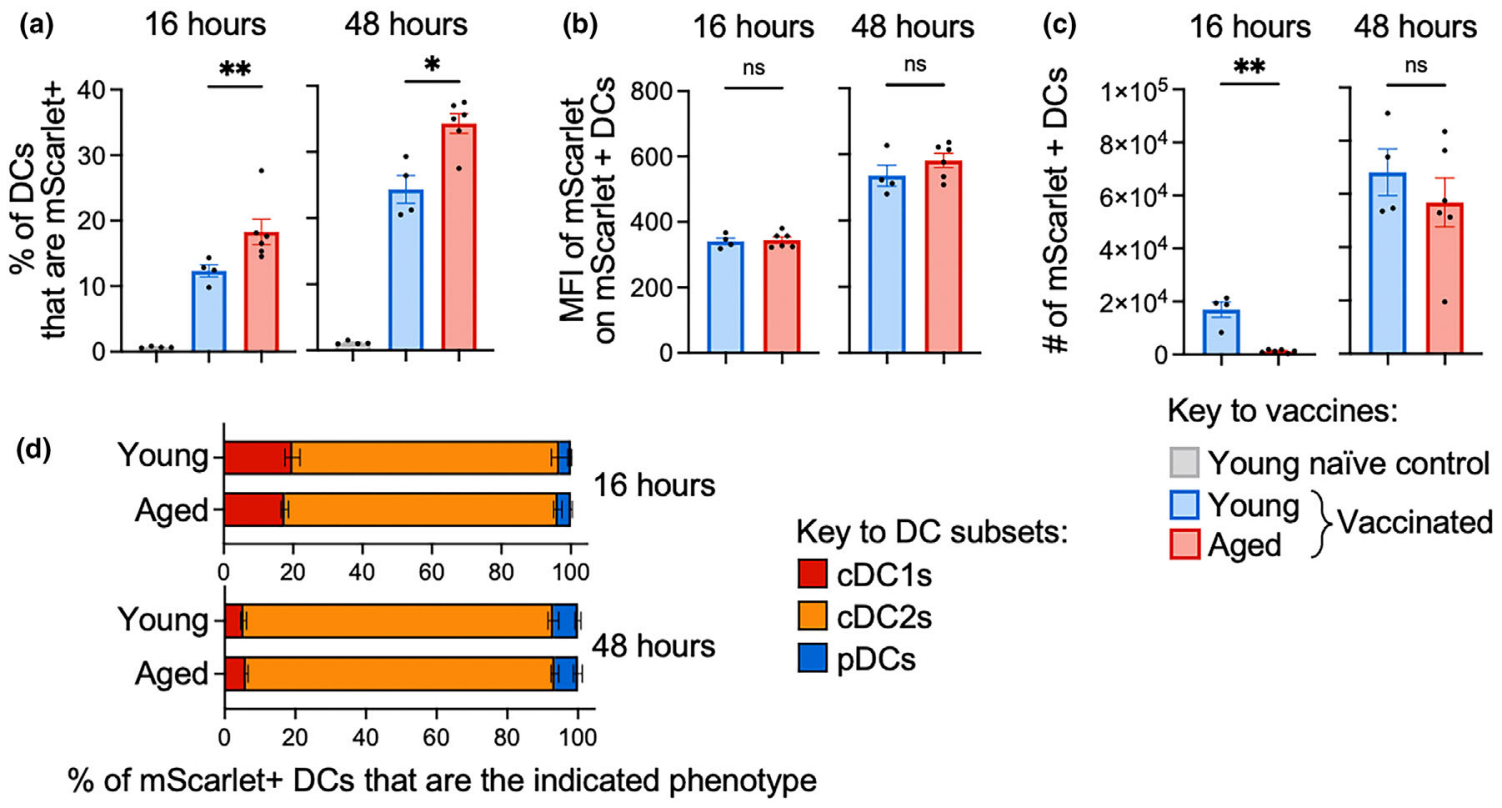
Aging increases the frequency of antigen expressing DCs after mRNA vaccination. **(a)** The frequency of DCs that are mScarlet^+^, **(b)** the MFI of mScarlet on mScarlet^+^ DCs, **(c)** absolute numbers of mScarlet^+^ DCs and **(d)** the frequency of mScarlet^+^ DC in dLN that are each subset at 16 and 48 h after mRNA vaccination in young and aged vaccinated mice. Symbols indicate mean, error bars indicate SEM. (*n* = 4 (young vaccinated) or 6 (aged vaccinated). ns indicates not significant, * indicates *P* ≤ 0.05, ** indicates *P* ≤ 0.01, *** indicates *P* ≤ 0.001, using a Mann–Whitney *U* test. Data are representative of at least 2 independent experiments.

We then evaluated how aging impacted antigen expression by DCs in the dLNs. The frequency of DCs that expressed mScarlet was significantly higher in aged as compared to young mice after vaccination at both 16 and 48 h after vaccination (Figure 7a), but the MFI of mScarlet staining within those DCs did not differ (Figure 7b). The absolute number of mScarlet^+^ DCs recovered from the dLNs was still substantially lower in aged as compared to young mice at 16 h after vaccination but was comparable by 48 h after vaccination (Figure 7c). When DC subset composition was assessed, markedly more mScarlet^+^ DCs were cDC2s (Figure 7d) as compared to total DCs (Figure 5d), particularly at 48 h after vaccination, which suggests that cDC2s preferentially make or retain antigen after mRNA vaccination. However, the frequencies of subsets within mScarlet^+^ DCs did not differ across young and aged mice (Figure 7d), which demonstrates aging does not alter the distribution of antigen to distinct DC subsets.

### Aging reduces CD86 expression on DCs

Finally, we evaluated how aging impacted DC activation before and after vaccination in the dLNs. In naïve mice, the frequency of DCs that expressed CD86 was significantly lower in aged as compared to young mice in the dLNs but was very low and equivalent across young and aged mice in the spleen (Supplementary figure 5a). In vaccinated mice, the frequency of DCs that expressed CD86 was equivalent in young and aged mice at 5 h after vaccination but significantly lower in aged as compared to young mice by 48 h after vaccination (Supplementary figure 5b, c). Similar age‐related patterns were observed for the MFI of CD86 on DCs (Supplementary figure 5d, e). This suggests that aging results in reduced DC activation in the dLN during both homeostasis and stimulus.

## DISCUSSION

In this study, we validated an aged mouse model of mRNA/LNP vaccination and used it to identify age‐related changes in adjuvancy mechanisms. Aging clearly led to a decline in mRNA vaccine immunogenicity, with decreased CD4 and CD8 T cell responses and B cell responses by all measures assessed. Aging also altered parameters associated with vaccine adjuvancy, perturbing kinetics of inflammatory cytokine production, reducing DC numbers, delaying DC recruitment to the dLN, increasing antigen loading in DCs in the dLN, and reducing DC activation.

The most striking age‐related deficit in adjuvancy was the drop in DC numbers in the dLNs, observed both prior to and early after mRNA vaccination. An age‐related reduction in DC number was also seen in the spleen, but reductions in the dLN were more dramatic, with an ~8.6‐fold reduction in the dLN as compared to a ~1.6‐fold reduction in the spleen. DC numbers in dLNs of young mice started to increase from 16 h after vaccination, while aged mice did not exhibit a marked increase until 48 h after vaccination. Our data therefore suggest that there is an age‐related defect in DC homeostasis that is particularly marked in LNs that drain peripheral tissues. This is a concern because muscle‐dLNs are the most relevant site for parenteral delivery of most vaccines and this deficit could therefore impact a wide variety of vaccines. In humans, it has been noted that skin‐dLNs undergo more age‐driven atrophy with larger reductions in total cell number than LNs draining mucosal sites.^36^ In mice, studies reported either modest declines or no difference in DC numbers in the spleen,^37^, ^38^ but we could not find reports of DC numbers specifically in mouse skin/muscle‐dLNs.

Based on our data and the literature, we suggest that the decline in DC numbers is caused by a decline in steady‐state DC migration to the dLN. Aged human and mouse DCs exhibit reduced inflammation‐driven migration rates and reduced expression of essential chemokine receptors such as CCR7.^33^, ^34^, ^35^, ^39^ The LN niche itself is also known to become defective with age, with a reduction in fibroblastic reticular cells that can be a source of CCL19 and CCL21 chemokines that recruit DCs via CCR7.^40^ We did not directly measure DC migration rates or expression of CCR7, CCL19, or CCL21, but the observed delay in DC influx into aged dLNs strongly supports a migratory defect. Markers of DC activation can also be informative for understanding DC dynamics in dLNs during homeostasis and activation. Splenic DCs in naïve mice had low levels of CD86 expression, reflecting that they are seeded directly from the blood, while DCs in dLNs had much higher CD86 expression, reflecting more dependence on DCs transiting from barrier sites in the skin. Expression of CD86 in naive dLNs was significantly lower in aged as compared to young mice, which suggests that reduced steady‐state activation of DCs may reduce migration to the dLN during homeostasis. Expression of CD86 in dLNs 48 h after vaccination was also significantly lower in aged as compared to young mice, which suggests reduced activation in response to the vaccine stimulus could contribute to reduced DC migration to the dLN. The mechanism driving reduced DC activation is unclear. It could be due to sensing of relevant PAMPs or DAMPs, as aging was seen to reduce surface or mRNA expression of TLR3, TLR7, and TLR8 and monocytes or DC subsets in other studies.^41^ However, reduced DC activation could also be due to altered DC‐intrinsic signaling downstream of key PRRs.^42^

Vaccine uptake was largely unaffected by aging, which is consistent with other studies tracking uptake of protein antigen that saw similar rates of uptake across young and aged mice.^35^ However, it was notable that aged mice had a significantly higher frequency of antigen‐bearing DCs as compared to young mice. This antigen was predominantly expressed by cDC2s in both young and aged mice. We speculate that this finding may reflect a retention of antigen in aged DCs, as aging is known to impede proteostasis.^43^ Delayed protein degradation could lead to more DCs containing intact antigen, but it could also undermine T cell priming if degradation of antigen and presentation of epitopes on MHCI and II are delayed or impaired. Unfortunately, we did not have access to appropriate methods to directly assess antigen processing and presentation with mRNA vaccines, although aging has been seen to impair processing of soluble antigen with human monocyte‐derived DCs.^44^ We also did not assess persistence of antigen beyond 48 h, which could impact on mRNA vaccine adjuvancy. mRNA template is known to be retained in the dLN for up to 30 days after vaccination,^45^ which likely drives the prolonged germinal center activity typically seen in vaccinees.^46^

Highly consistent, age‐related shifts in expression of early inflammatory cytokines were seen in both dLNs and the serum. Production of IL‐1α and IL‐6 was increased or accelerated, TNF and IFNβ were unchanged, and IFNγ and MCP‐1 were decreased or delayed in the dLNs and/or sera. The patterns of cytokine production (increased, unchanged or decreased) in young vs aged mice were highly consistent across local and systemic compartments, underlining the robustness of this dataset. Increases in IL‐1α and IL‐6 are characteristic of inflammageing, where the immune system becomes hyper‐inflamed with increasing age,^47^, ^48^ so hyper‐responsiveness with these cytokines may reflect the hyper‐inflamed aged environment. Changes in any single cytokine were not predictive of T cell or B cell immunogenicity, but instead aging clearly perturbs the kinetics of production for multiple inflammatory cytokines relative to one another. This loss of synchronization of inflammatory cytokines locally and systemically could itself contribute to the age‐related decline in immunogenicity seen with mRNA vaccination. Combined with the delay in DC recruitment to dLNs, we propose that aging results in a loss of synchronization between early inflammatory responses and antigen presentation in the dLN.

One key limitation of our study is that the model may underestimate the impact of aging on IL‐1β as a mediator of vaccine adjuvancy. IL‐1β is induced by activation of the NOD‐like receptors and is one of the main cytokines produced *in vitro* and *in vivo* with humans after mRNA vaccination.^49^ However, we and others have observed that IL‐1β is not made at high levels in C57BL/6 mice after mRNA vaccination. Indeed, mice are known to express extremely high levels of the IL‐1 receptor antagonist (IL‐1Ra), which blocks amplification of IL‐1β production after mRNA vaccination.^49^ As a result, wildtype mouse models can underestimate IL‐1β‐driven impacts of vaccine adjuvancy. To mitigate this, our findings should be validated either with IL‐1Ra deficient mice,^49^ with *in vitro* human models such as the LN explant system,^50^ or with *in vivo* human models using fine needle aspirates of LNs from vaccinees.^51^ Another caveat is that aging is known to cause T cell‐intrinsic defects in proliferation and cytokine production.^30^ Novel vaccine strategies might be able to improve T cell‐extrinsic factors related to adjuvancy that limit adaptive immunity in aged systems, but defects intrinsic to adaptive immune cells may still limit vaccine efficacy. In addition, we have not examined B cell‐specific vaccine mechanisms, such as germinal center reactions or memory cell formation, as our broader work is focused on mechanisms of T cell immunity and other groups have published extensive analyses of B cell immunity with vaccines.^52^, ^53^ Finally, our work identified a significant limitation of AIM assays in studies of aging. Aged mice had very high baseline expression of activation‐induced markers, which impaired our ability to accurately measure more modest antigen‐specific T cell responses using this method. We thereby propose that ICS assays are a superior metric for antigen‐specific T cell responses in an aged mouse model and caution that this may also extend to studies of older humans.

Despite these limitations, our study supports several strategies to improve vaccine responses in older people: increase DC numbers, target DCs in the spleen, promote DC migration, promote antigen processing, or re‐synchronize innate cytokines. DC numbers could be increased using Fms‐like tyrosine kinase‐3 ligand (Flt3L), which can expand DCs *in vivo* in both mice and humans.^54^, ^55^ Flt3L has been administered prior to vaccination to augment tumor‐specific adaptive responses in a phase II clinical trial of a cancer vaccine,^56^ but its impact on DC numbers in dLNs and adaptive immunity should be assessed specifically in older individuals. DCs are more abundant in the aged spleen and could be better targeted by simply changing the route of mRNA vaccine delivery from intramuscular to intravenous. An mRNA vaccine currently in development for pancreatic cancer is delivered intravenously,^6^, ^7^ and our data would predict that this could be a better route, especially as many pancreatic cancer patients are older. DC migration to dLNs could be increased with DC hyperactivating adjuvants like 1‐palmitoyl‐2‐glutaryl phosphatidylcholine (PGPC). PGPC is a potent trigger for IL‐1β production and was seen to enhance CCR7 expression and migration capacity in aged mouse bone marrow‐derived DCs and monocyte‐derived DCs from older donors.^39^ Current mRNA/LNP vaccine formulations already trigger robust IL‐1β production in humans and human cells, presumably due to the action of ionizable lipids with the cell membrane.^23^ However, for older patients, the use of LNPs with ionizable lipids that trigger stronger IL‐1β responses may support more DC migration and thereby support more robust adaptive immunity. Antigen processing could be increased by coadministration of mRNA vaccines with agents that upregulate proteolysis such as spermidine. Indeed, spermidine is currently being trialed as a supplement with an inactivated influenza vaccine in older patients (NCT05421546), and our data suggest that this approach is also well‐rationalized for mRNA vaccines. Finally, re‐synchronization of aged innate cytokine responses would be challenging to achieve, but one potential target could be to prevent or reduce inflammageing prior to vaccination. This could give signaling pathways the chance to reset and reduce the early burst in IL‐1α and IL‐6 production.

Altogether our study shows that an aged mouse model recapitulates the age‐related deficits observed in adaptive immune responses after mRNA vaccination in older humans. In the mouse model, aging reduces DC numbers and limits recruitment to dLNs, increases early antigen load, reduces DC activation, and perturbs early cytokine expression. These insights can be used to support the design of strategies to specifically improve mRNA vaccination in older people.

## METHODS

### Mice

Female C57BL/6 mice were obtained from the Animal Resources Centre, WA, Australia, and Ozgene, WA, Australia. Young mice were received at 6 weeks of age, aged mice were received as ex‐breeders at 9 months old (mo), and all mice were housed in specific‐pathogen‐free conditions in the RMIT Animal Facility at RMIT University, Bundoora West campus until use in our study. Young mice were 2–5 months, and aged mice were 18–22 months. Mice were examined for gross abnormalities (tumors and enlarged livers, spleens, or LNs) and excluded from analyses if these were evident. All work was performed in accordance with local legislation and institutional regulations after review and approval by the RMIT University Animal Ethics Committee under ethics application ID #23208.

### Vaccines

mRNA/LNP vaccines were generated at the Monash Institute of Pharmaceutical Sciences (MIPS). All LNPs used a “Pfizer/BioNTech”‐like formulation comprising cholesterol (Sigma‐Aldrich, St Louis, MA, USA), DSPC (Avanti Polar Lipids Inc, Alabaster, AL, USA), Methoxypolyethyleneglycoloxy(2000)‐N,N‐ditetradecylacetamide (ALC‐0159) (MedChemExpress, Monmouth Junction, NJ, USA) and [(4‐hydroxybutyl)‐azanediyl]‐di‐(hexane‐6,1‐diyl)‐bis‐(2‐hexyldecanoate) (ALC‐0315) (MedChemExpress). All mRNA sequences were optimized for minimal uridine content, N1‐methyl‐pseudoUTP was used instead of UTP, and co‐transcriptional capping (Cap1, Trilink) was performed during *in vitro* transcription, followed by cellulose purification. Specific LNP and mRNA production conditions, including UTRs and methods are detailed in our prior study.^57^ Three vaccines were used in this study: (i) an mRNA/LNP vaccine encoding the full‐length Spike protein from the SARS‐CoV‐2 Wuhan variant, (ii) an mRNA/LNP vaccine encoding the full‐length luciferase protein, loaded with DiD lipid dye (Invitrogen, Waltham, MA, USA) added at 2% by mole to the pre‐LNP formulation, and (iii) an mRNA/LNP vaccine encoding the mScarlet fluorescent protein. For each prime or boost vaccination, a dose of 5 μg in 100 μL was administered intramuscularly into the gluteal muscles of each hind leg (i.e. 2 injections of 50 μL).

### Intracellular cytokine staining (ICS) assay

Spleens were harvested, mechanically disrupted through a 70 μm cell strainer, and then red blood cells were lysed using ACK Lysing Buffer (Invitrogen) as per manufacturers' instructions. Approximately 1 × 10^6^ isolated leukocytes were stimulated with anti‐CD28 monoclonal antibody (mAb) (clone 37.51; BD Pharmingen; 1 μg/mL) and either (i) no peptide as a background control, (ii) PepTivator^®^ SARS‐CoV‐2 Prot_S containing overlapping 15‐mer peptides to activate CD4 T cells along the full‐length of the SARS‐CoV‐2 spike protein (Miltenyi Biotec, Bergisch Gladbach, Germany; 1 μg/mL), or (iii) a SARS‐CoV‐2 spike protein‐derived peptide that is immunodominant for CD8 T cells in C57BL/6 mice (residues 539–546, VNFNFNGL; Genscript, Jiangsu, China; 1 μg/mL). Stimulation media also contained anti‐CD107a FITC mAb (clone 1D4B; BD Pharmingen, New Jersey USA; 1:200 dilution) and GolgiStop (BD Biosciences, New Jersey USA; 1:1000 dilution). Gating for cytokines was based on a positive control where the stimulation media contained anti‐CD3 mAb (clone 145‐2C11; BD Pharmingen; 1 μg/mL) and fluorescence minus one (FMO) controls. Cells were incubated for 5 h at 37°C and 5% CO_2_ then stained for surface markers (LIVE/DEAD Fixable Near InfraRed Viability Dye (Invitrogen), anti‐CD8‐v450 (clone 53–6.7; Tonbo, California, USA), anti‐CD4‐AF700 (clone GK1.5; BioLegend, San Diego, CA, USA), anti‐CD3‐PerCP‐Cy5.5 (clone 145‐2C11; BD Pharmingen)). Cells were fixed and permeabilized with the BD CytoFix/CytoPerm kit (BD Biosciences) as per manufacturer's instructions, then stained for intracellular markers (anti‐IFN‐γ‐APC (clone XMG1.2; BD Pharmingen), anti‐IL‐2‐PE (clone JES6‐5H4; BD Pharmingen), anti‐TNF‐PE‐Cy7 (clone MP6‐XT22; BD Pharmingen)).^58^ Sample gating trees are provided in Supplementary figure 1a. Reported values are background subtracted using no peptide controls.

### Activation‐induced marker (AIM) assay

Spleens were harvested and processed as described above. Approximately 1 × 10^6^ isolated leukocytes were stimulated with either (i) no peptide as a background control, (ii) PepTivator^®^ SARS‐CoV‐2 Prot_S peptide pool for CD4 T cells as above (Miltenyi Biotec; 1 μg/mL), or (iii) a SARS‐CoV‐2 spike protein‐derived peptide for CD8 T cells as above (Genscript; 1 μg/mL). Stimulation media also contained anti‐CD154 PE mAb (clone MR1; Tonbo; 1:1000 dilution). Gating for activation‐induced markers was based on stimulation with anti‐CD3 mAb (clone 145‐2C11; 1 μg/mL) and FMO controls. Cells were incubated for 16 h at 37°C and 5% CO_2_, then stained for surface markers (LIVE/DEAD Fixable Near InfraRed Viability Dye (Invitrogen), anti‐CD8‐v450 (clone 53–6.7; Tonbo), anti‐CD4‐AF700 (clone GK1.5; BioLegend), anti‐CD3‐PerCP‐Cy5.5 (clone 145‐2C11; BD Pharmingen), anti‐CD25‐FITC (clone PC61.5; Tonbo), anti‐CD69‐BV605 (clone H1.2F3; BD Pharmingen), anti‐OX40‐APC (clone OX‐86; Tonbo)). Sample gating trees are provided in Supplementary figure 1b. Reported values are background subtracted using no peptide controls.

### Enzyme‐linked immunosorbent assay (ELISA)

Spike protein or RBD peptide (1 μg/mL) diluted in 0.1 M carbonate coating buffer was adsorbed to Nunclon MaxiSorp 96 well plates (ThermoFisher, Waltham, MA, USA) overnight. Plates were washed 3 times with wash buffer (0.05% Tween20/PBS), blocked with 100 μL of assay diluent (10% FCS/PBS) for 2 h at room temperature (RT), then washed 3 times again. Sera from mice was first diluted 1:10 and then prepared as a 1:6 dilution series in assay diluent, 100 μL was added to the plates, then incubated for 2 h at RT. A positive control sample (pooled sera from young, vaccinated mice at peak) was run on each plate to control for variation in processing and calculate endpoints across plates. Plates were washed 3 times, 100 μL of Biotinylated Goat Anti‐Mouse IgG antibody (subclasses 1 + 2a + 2b + 3) (Jackson ImmunoResearch, Philadelphia, PA, USA; 1:20000) was added and incubated for 1 h at RT. Plates were washed 3 times, 100 μL of goat anti‐mouse IgG horse radish peroxidase (eBiosciences, San Diego, CA, USA; 1:1000) was added and incubated for 1 h at RT. Plates were washed 6 times, 100 μL of 3,3′,5,5′‐Tetramethylbenzidine TMB Substrate Reagent (BD Biosciences) was added and incubated for 5 min at RT in the dark. Finally, 100 μL of ELISA Stop Solution (2 N H_2_SO_4_) was added and absorbance was measured at 450 nm on a CLARIOStar‐BMG‐Plate Reader (BMG Labtech, Ortenberg, Germany). Endpoint titer of IgG antibody was measured by calculating the endpoint absorbance for each plate (15% of max absorbance of the positive control) then interpolating from sigmoidal curve fit using GraphPad Prism 10.4.1 software.^59^

### Surrogate virus neutralization titer (sVNT) assay

Mouse sera were heat‐inactivated at 56°C for 45 min then prepared as a dilution series. MagPlex‐Avidin microspheres (Luminex, Genk, Belgium) were coated with AviTag‐biotinylated RBD proteins from SARS‐CoV‐2 variants at 5 μg per 1 million beads. Sera serial dilutions were mixed with coated microspheres (600 beads per antigen) and incubated for 1 h at 37°C with 800 rpm agitation. Fifty μL of PE‐conjugated human ACE2 (1 μg/mL; GenScript) was added and incubated for 30 min at 37°C with agitation. Beads were washed twice with 1% bovine serum albumin in PBS, and samples were acquired using a MAGPIX reader (Luminex) and expressed as half‐maximal inhibitory dilution (sVNT50).^57^

### Legendplex assay

Sera and dLNs were harvested at 0, 5, 16 and 48 h after vaccination. dLNs were processed into lysates by mechanical disruption with a sterile plastic pestle in sterile eppendorfs containing 300 μL of cRPMI. Lysates were transferred into a 96‐well U bottom plate and incubated for 6 h at 37°C and 5% CO_2_. Solid material was pelleted by centrifugation, and lysate supernatant was removed and frozen at −80°C. Sera and dLN lysate supernatants were then used in the LEGENDplex™ Mouse Inflammation Panel Assay (BioLegend, CA, USA) according to the manufacturer's instructions.

### DC assays

Inguinal and popliteal LNs were harvested and pooled for each mouse at the indicated timepoint after vaccination. Tissues were mechanically homogenized with scissors before enzymatic digestion as described previously.^58^ To control for variation in sample processing, 200 000 Accucheck beads (Invitrogen) were added. Cells were incubated with Fc block (clone 2.4G2; Tonbo; 1:50 dilution), stained with either anti‐CD11c‐PE (with DiD‐loaded mRNA/LNPs; clone HL3; BD Pharmingen) or anti‐CD11c‐APC (with mScarlet‐encoding mRNA/LNPs; clone N418; Tonbo), then enriched by fluorophore^+^ positive selection according to the manufacturer's instructions (Miltenyi Biotec) Cells were surface stained with the DC panel (LIVE/DEAD Fixable AquaBlue Viability Dye (Invitrogen), anti‐NK1.1‐FITC (clone PK136; BD Pharmingen), anti‐CD19‐FITC (clone 1D3; BD Pharmingen), anti‐CD3‐PerCP‐Cy5.5 (clone 145‐2C11; BD Pharmingen), anti‐B220‐PE‐Cy7 (clone RA3‐6B2; Tonbo), anti‐CD8‐v450 (clone 53–6.7; Tonbo), anti‐CD11b‐APC‐Cy7 (clone M1/70; Tonbo), and anti‐SIRPα‐AF647 (clone P84; eBiosciences)). The complete sample was acquired on a flow cytometer, then Accucheck beads were gated on and used to quantitate the total number of recovered DCs. Sample gating trees are provided in Supplementary figure 1c, d.

### Data and statistical analysis

All flow cytometry data were acquired on a BD LSRFortessa flow cytometer (BD Biosciences), analyzed using FlowJo version 10.10 and graphed in GraphPad Prism version 10.4.1, although LegendPlex data were analyzed using Qognit online software. All data with logarithmic distribution (antibody titers or half‐lifes, neutralization titers, MFI) were transformed (log_10_) before plotting and statistical analyses were performed. Naïve young mice were plotted to define background in some of our assays, but statistical significance was assessed between young and aged vaccinated mice using statistical tests as indicated in each figure legend. Data from this study are available from the corresponding author upon reasonable request.

## CONFLICT OF INTEREST

We declare no conflict of interest.

## Supporting information


Supplementary figure 1.
**Supplementary figure 2**.
**Supplementary figure 3**.
**Supplementary figure 4**.
**Supplementary figure 5**.

## Data Availability

The data that support the findings of this study are available from the corresponding author upon reasonable request.
