# Supplementary material for: Age‐related differences in mRNA vaccine immunogenicity and adjuvancy
Source: Immunol Cell Biol. 2026 Mar 12;104(4):400–14. doi: 10.1111/imcb.70102 (PMC13071135; doi:10.1111/imcb.70102)
Supplement: Supplementary file 1 — Supplementary figure 1. Supplementary figure 2. Supplementary figure 3. Supplementary figure 4. Supplementary figure 5. [file IMCB-104-400-s001.pdf]

SUPPLEMENTARY MATERIALS FOR AGE-RELATED DIFFERENCES IN mRNA VACCINE IMMUNOGENICITY AND ADJUVANCY

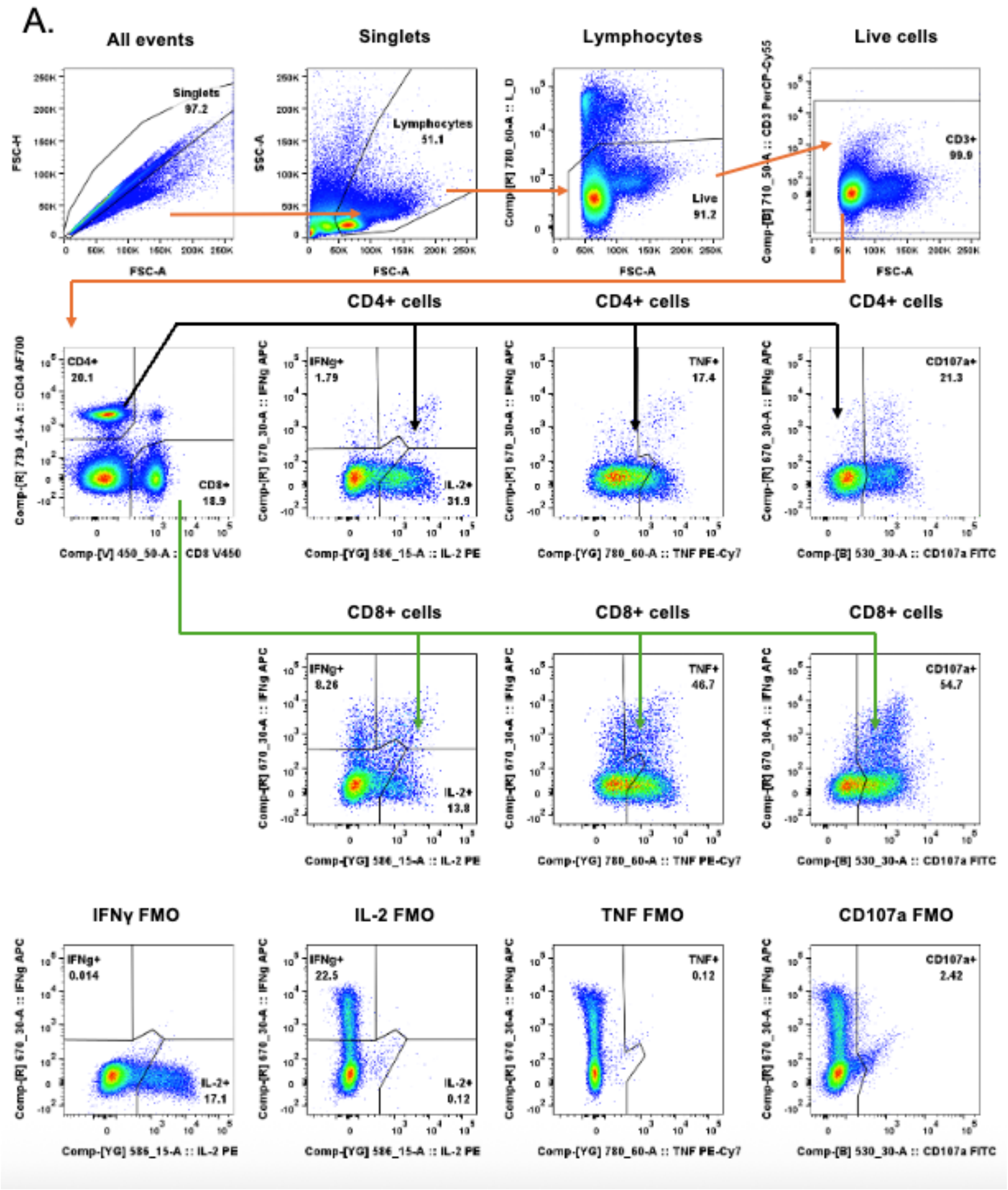

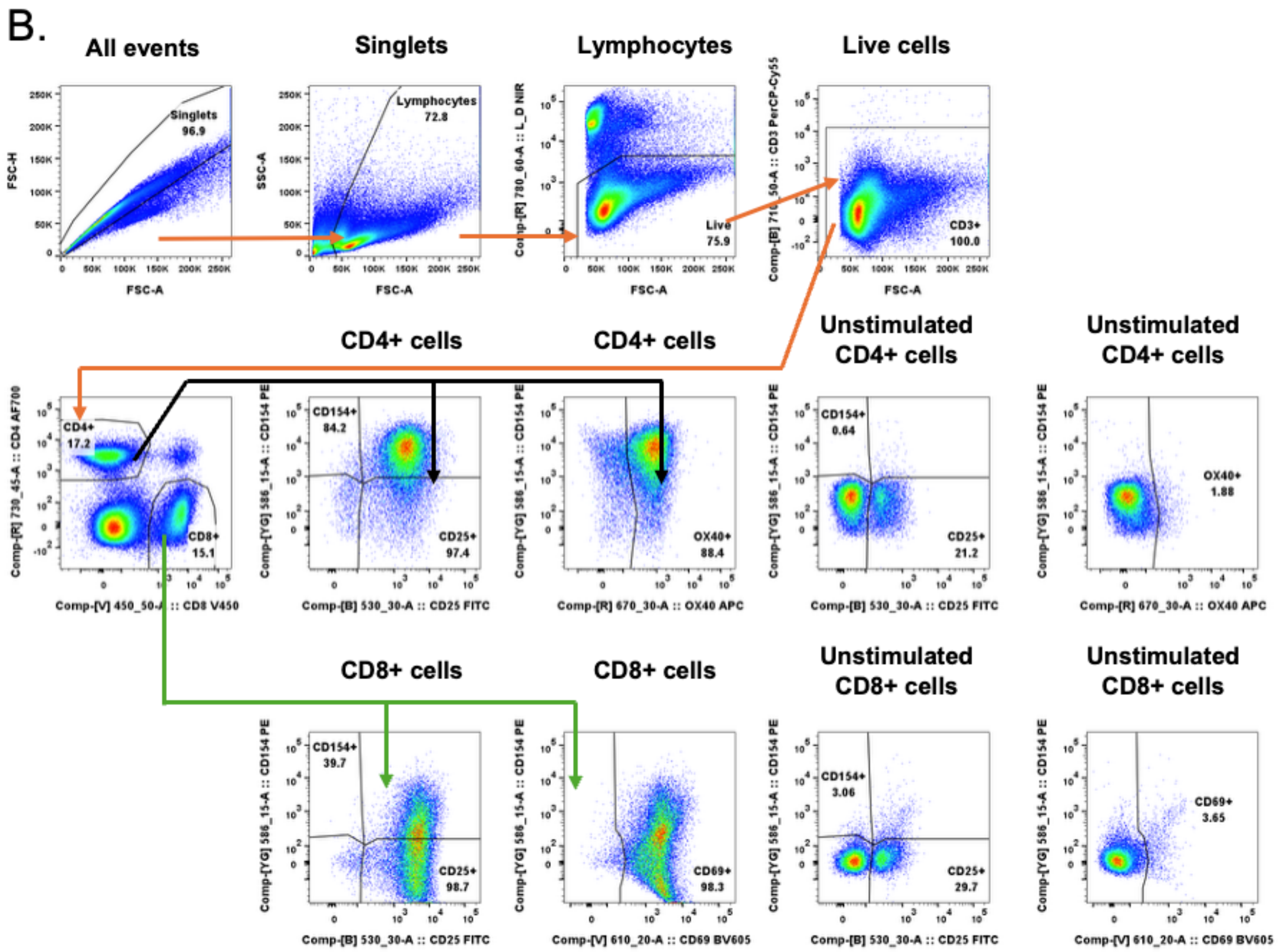

B.
All events
Singlets
Lymphocytes
Live cells
Singlets 96.9
Lymphocytes 72.8
Live 75.9
CD3+ 100.0
FSC-H
SSC-A
FSC-A
Comp-[R] 780_60-A :: L_D NIR
Comp-[B] 710_50-A :: CD3 PerCP-Cy55
CD4+ cells
CD4+ cells
Unstimulated CD4+ cells
Unstimulated CD4+ cells
CD4+ 17.2
CD8+ 15.1
CD154+ 84.2
CD25+ 97.4
OX40+ 88.4
CD154+ 0.64
CD25+ 21.2
OX40+ 1.88
Comp-[R] 730_45-A :: CD4 AF700
Comp-[V] 450_50-A :: CD8 V450
Comp-[YG] 586_15-A :: CD154 PE
Comp-[B] 530_30-A :: CD25 FITC
Comp-[R] 670_30-A :: OX40 APC
CD8+ cells
CD8+ cells
Unstimulated CD8+ cells
Unstimulated CD8+ cells
CD154+ 39.7
CD25+ 98.7
CD69+ 98.3
CD154+ 3.06
CD25+ 29.7
CD69+ 3.65
Comp-[V] 610_20-A :: CD69 BV605

C.

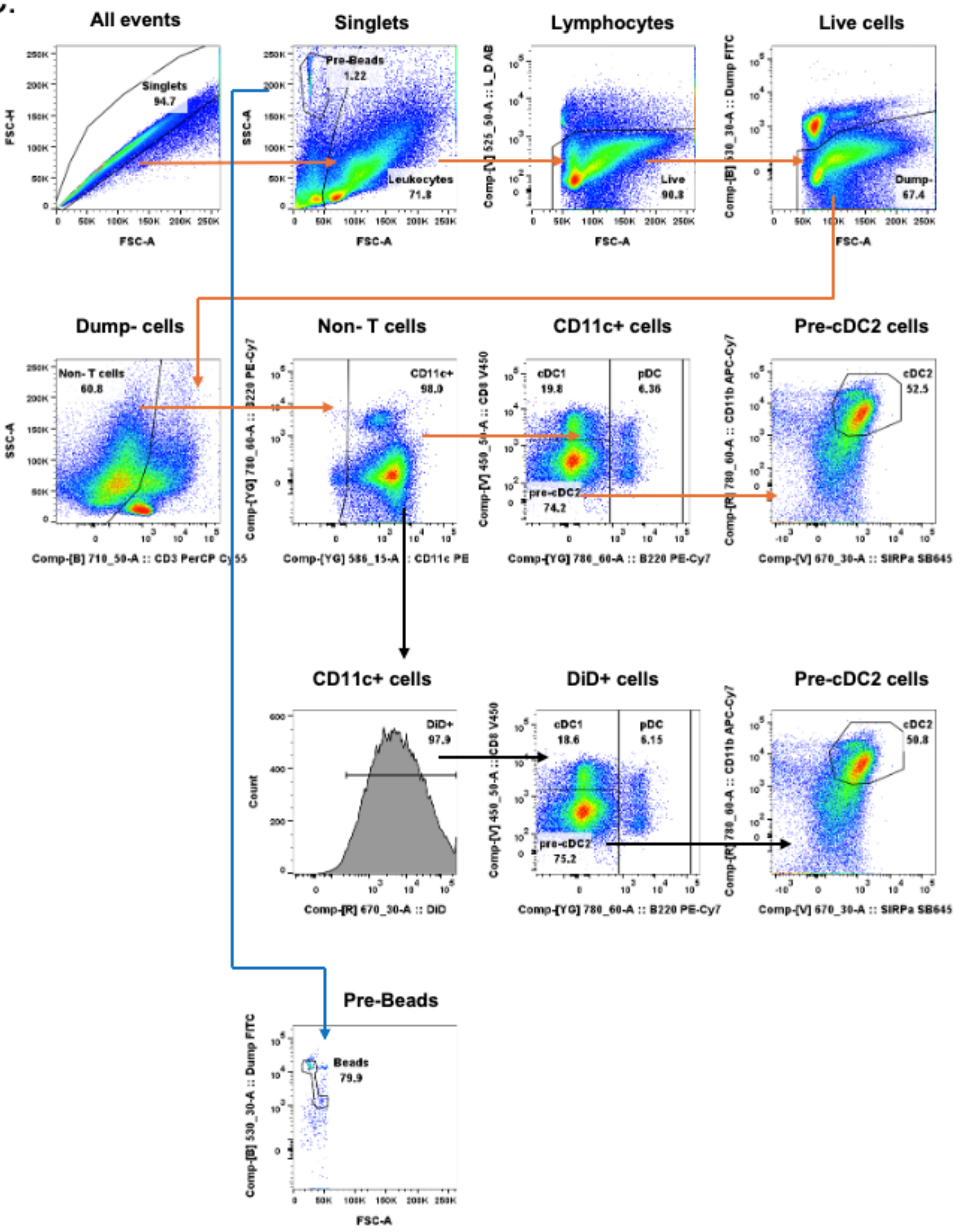

All events
Singlets
94.7
FSC-H
FSC-A
Singlets
Pre-Beads
1.22
Leukocytes
71.8
SSC-A
FSC-A
Lymphocytes
Comp-[V] 525_50-A :: L_D AB
Live
90.8
FSC-A
Live cells
Comp-[B] 530_30-A :: Dump FITC
Dump-
67.4
FSC-A
Dump- cells
Non- T cells
60.8
SSC-A
Comp-[B] 710_50-A :: CD3 PerCP Cy55
Non- T cells
CD11c+
98.0
Comp-[YG] 780_60-A :: B220 PE-Cy7
Comp-[YG] 586_15-A :: CD11c PE
CD11c+ cells
cDC1
19.8
pDC
6.36
pre-cDC2
74.2
Comp-[V] 450_50-A :: CD8 V450
Comp-[YG] 780_60-A :: B220 PE-Cy7
Pre-cDC2 cells
cDC2
52.5
Comp-[R] 780_60-A :: CD11b APC-Cy7
Comp-[V] 670_30-A :: SIRPa SB645
CD11c+ cells
DiD+
97.9
Count
Comp-[R] 670_30-A :: DiD
DiD+ cells
cDC1
18.6
pDC
6.15
pre-cDC2
75.2
Comp-[V] 450_50-A :: CD8 V450
Comp-[YG] 780_60-A :: B220 PE-Cy7
Pre-cDC2 cells
cDC2
50.8
Comp-[R] 780_60-A :: CD11b APC-Cy7
Comp-[V] 670_30-A :: SIRPa SB645
Pre-Beads
Beads
79.9
Comp-[B] 530_30-A :: Dump FITC
FSC-A

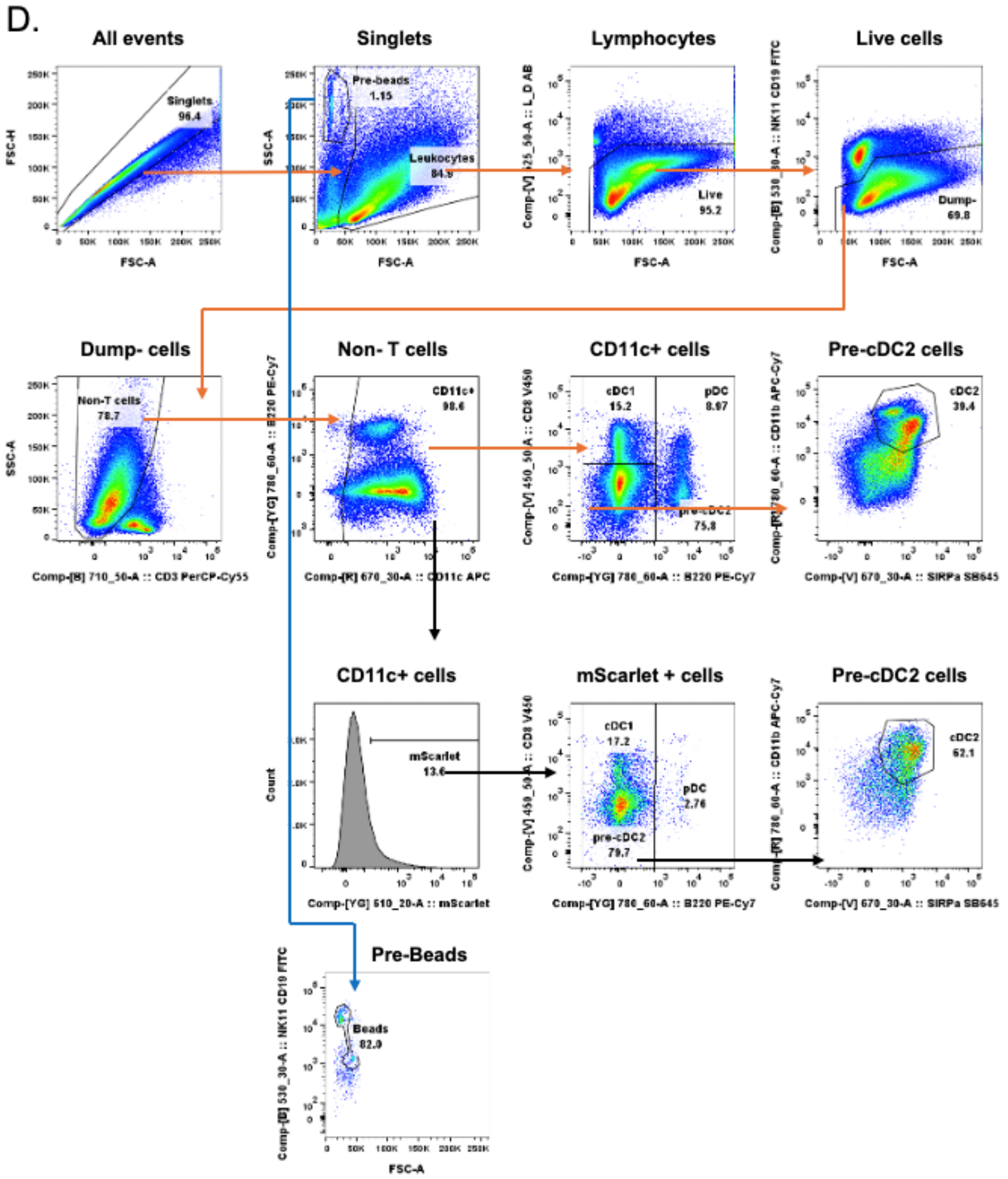

Supplementary figure 1: Sample gating trees. **A)** Gating for the ICS assay. **B)** Gating for the AIM assay. **C)** Gating for DC profiling with a DiD-loaded vaccine. **D)** Gating for DC profiling with an mScarlet-encoding vaccine. Note that CD3 gates for ICS and AIM assays included all cells. These assays activate T cells to trigger cytokine production and activation marker expression, but activated T cells downregulate CD3 expression substantially- a stringent CD3 gate would omit the most activated T cells.

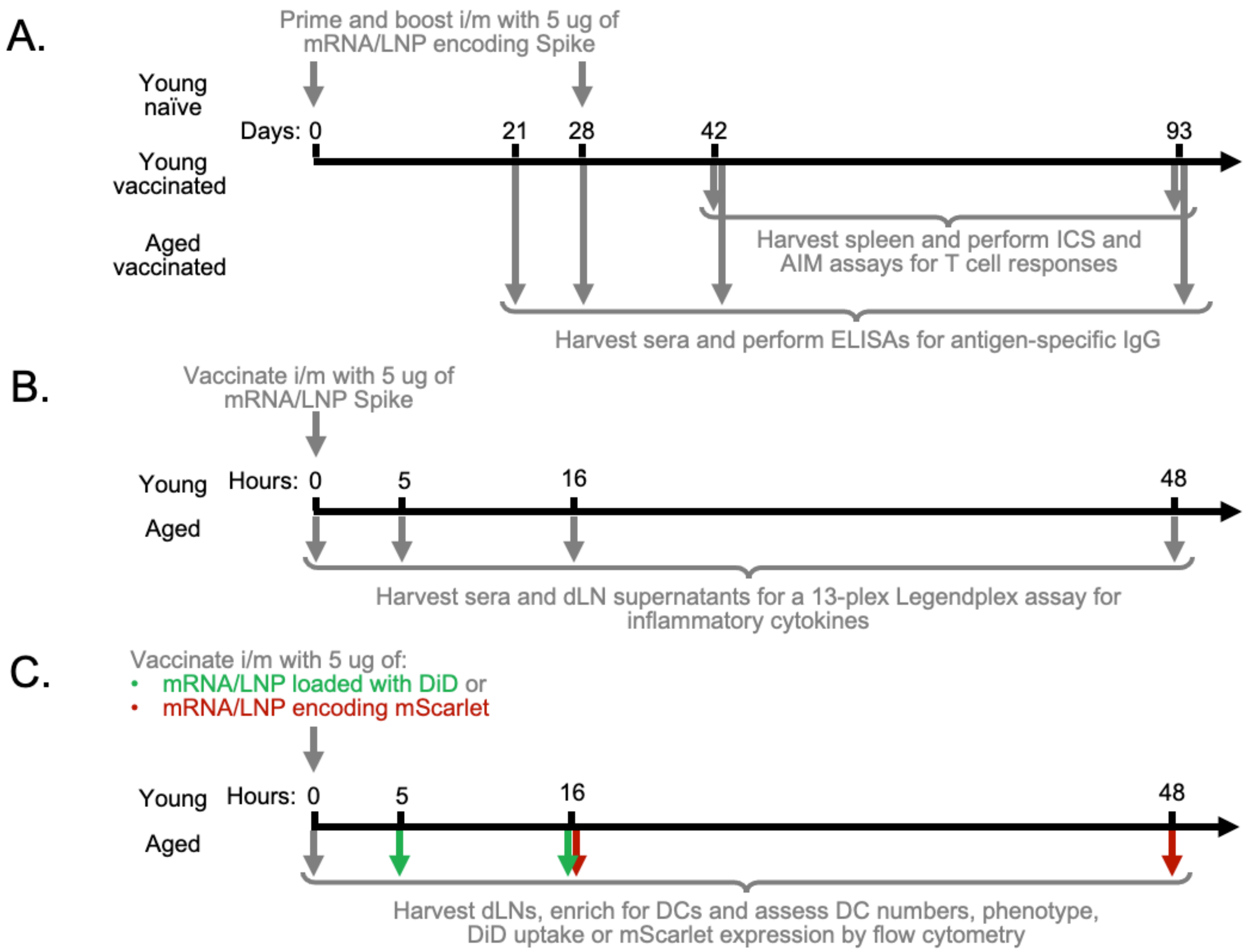

Supplementary figure 2: Schematics of experimental design. **A)** On day 0 and day 28, 5 ug of mRNA vaccine encoding the Wuhan-variant-derived, full-length spike protein was injected in each hind leg of young (<5 mo) or aged (>18 mo) mice. To assess antigen-specific T cell responses, splenocytes were harvested on days 42 and 93 to perform ICS and AIM assays. To assess antigen-specific B cell responses, sera was collected on days 21, 28, 42 and 93 to perform ELISA assays. Samples from young naïve mice were used to establish background in all assays. **B)** At hour 0, 5 ug of mRNA vaccine encoding the Wuhan-variant-derived, full-length spike protein was injected in each hind leg of young (<5 mo) or aged (>18 mo) mice. To assess local inflammatory markers, dLNs were harvested at 0, 5, 16 and 48 hours after vaccination, homogenised in RPMI, cultured for 5hrs and then supernatants were harvested for use in the Legendplex 13-plex mouse inflammation assay. To assess systemic inflammatory markers, sera were harvested at 0, 5, 16 and 48 hours after vaccination for use in the Legendplex 13-plex mouse inflammation assay. **C)** At hour 0, 5 ug of mRNA vaccine either i) loaded with DiD lipid dye or ii) encoding the mScarlet fluorescent protein was injected in each hind leg of young (<5 mo) or aged (>18 mo) mice. To assess DC numbers, phenotype, activation, vaccine uptake or antigen expression, dLNs were harvested at hours 0, 5, 16, and 48 after vaccination, DCs were magnetically enriched and parameters were assessed by flow cytometry. Vaccine uptake and antigen expression was measured by frequency and MFI of DiD+ DCs and mScarlet+ DCs respectively.

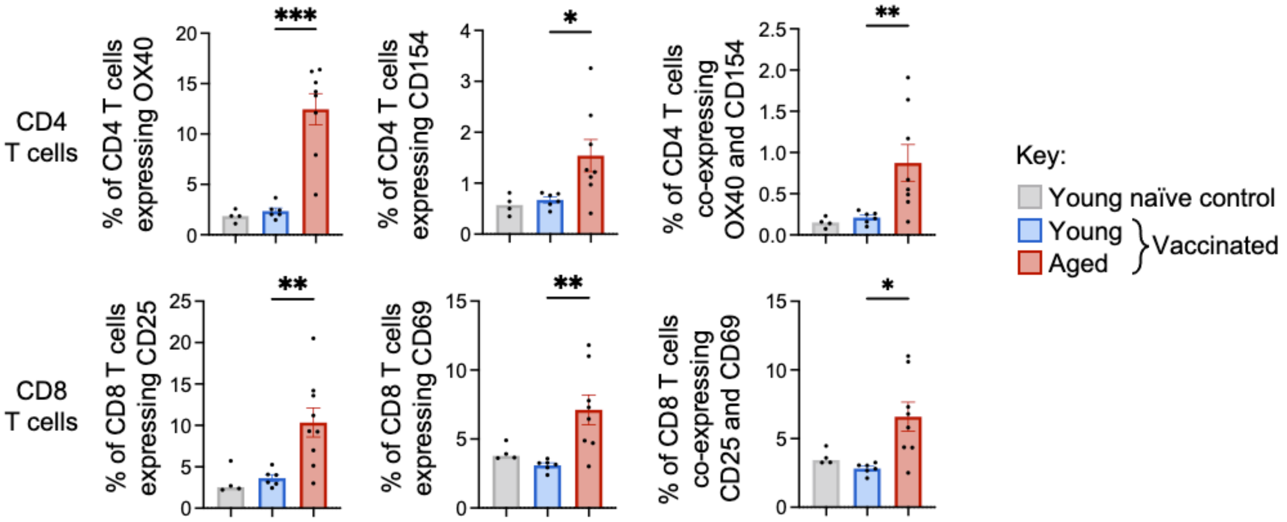

Supplementary figure 3: Ageing causes high background in AIM assays and reduces their sensitivity. Expression or co-expression of indicated activation-induced markers on unstimulated CD4 and CD8 T cells from young and aged mice at peak after vaccination. Bars indicate mean, error bars indicate SEM, and symbols indicate individual mice (n=4 (young naïve), 6 (young vaccinated) or 10 (aged vaccinated)). ns indicates not significant, * indicates $p \leq 0.05$, ** indicates $p \leq 0.01$, *** indicates $p \leq 0.001$, using a Mann-Whitney U test. Data are representative of 2 independent experiments.

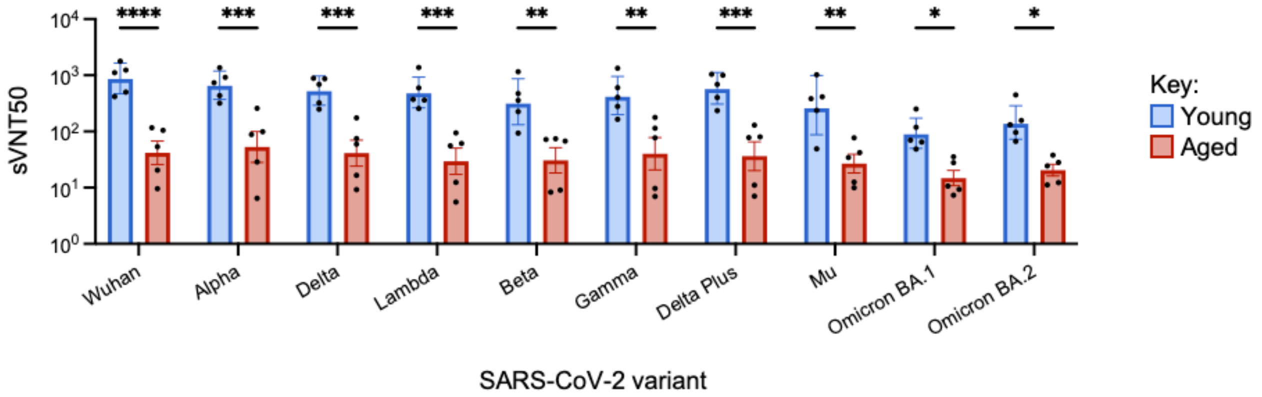

Supplementary figure 4: Ageing decreases surrogate viral neutralisation titres after mRNA vaccination. Half-maximal inhibitory dilution (sVNT50) values were determined in a multiplex RBD bead assay, which measures the relative ability of sera harvested from young or aged mice at memory (day 93) to neutralise binding of variant RBDs to ACE2. Bars indicate the mean, error bars indicate SEM, and symbols indicate individual mice (n= 5 (young vaccinated) or 5 (aged vaccinated)). ns indicates not significant, * indicates $p \leq 0.05$, ** indicates $p \leq 0.01$, **** indicates $p \leq 0.0001$, using a two-way ANOVA test with Sidak's correction for multiple comparisons.

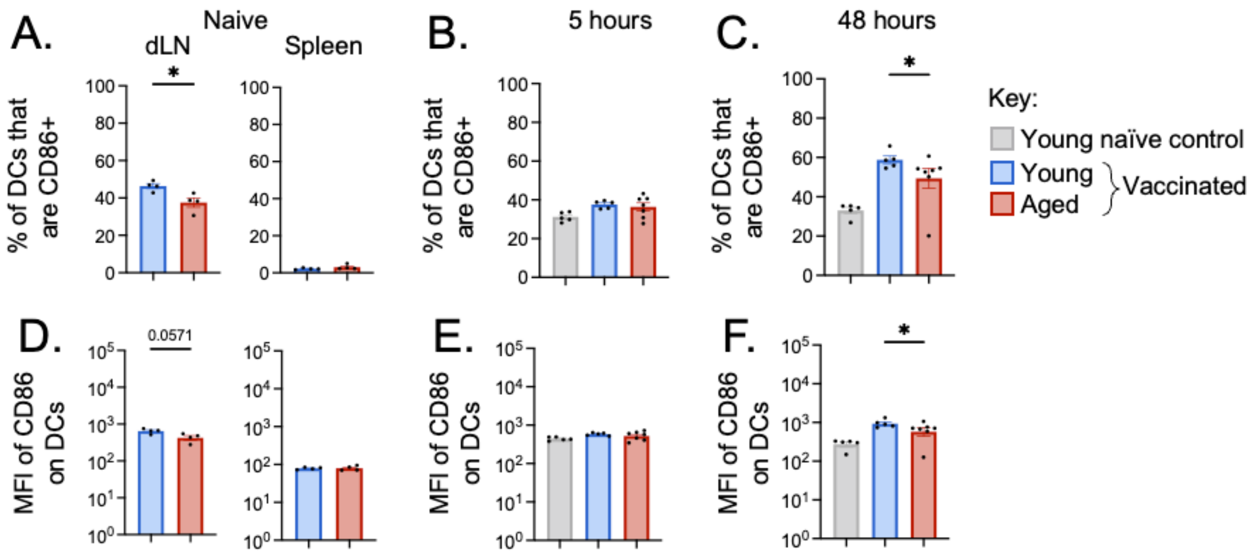

Supplementary figure 5: Ageing decreases expression of CD86 on DCs in the dLN before and after vaccination. The frequency of DCs expressing CD86 from **A)** naïve dLNs or spleen, **B)** dLNs 5 hours after vaccination or **C)** dLNs 48 hours after vaccination. The median fluorescence intensity (MFI) of CD86 on DCs from **D)** naïve dLNs or spleen, **E)** dLNs 5 hours after vaccination or **F)** dLNs 48 hours after vaccination. Bars indicate mean, error bars indicate SEM, and symbols indicate individual mice (n=4 (young naïve), 6 (young vaccinated) or 10 (aged vaccinated)). ns indicates not significant, * indicates $p \leq 0.05$, ** indicates $p \leq 0.01$, *** indicates $p \leq 0.001$, using a Mann-Whitney U test.
